# Development of uniform food information –the case of front of package nutrition labels in the EU

**DOI:** 10.1186/s13690-022-00915-1

**Published:** 2022-07-22

**Authors:** Federico Bottari, Cecilia Mark-Herbert

**Affiliations:** 1 Department of Molecular Sciences, Swedish University of Agricultural Sciences, Via degli Ulivi 135 A, 55047 Seravezza, Italy; 2 Department of Forest Economics, Swedish University of Agricultural Sciences, P.O. Box 7060, 750 07 Uppsala, Sweden

**Keywords:** Farm to fork strategy, Legislative process, Lobbying, Standard, Stakeholder management

## Abstract

The current malnutrition epidemic calls for actions. Current practices in the EU show a variety of communication efforts but the international character of food markets call for a harmonized language. The aim of the project is to identify the themes in the on-going debate regarding the development of a single front-of-package nutrition label in the European Union. A case study approach was used, focusing on the positions of different key stakeholders in Sweden and Italy. Overarching EU-perspectives, European Commission and European Council of Ministries were also included. Collected data from semi-structured interviews and strategic documents were used in a thematic content analysis. The results show that the stakeholders are influencing the process towards contradicting outcomes. Different stakeholders argue for opposing ideal labelling schemes, while still agreeing on the need for a harmonization. Major disagreements arise on whether the label should be voluntary or not, based on portion or 100 g and on the ideal label design. Stakeholders’ positions depend on food system role and previous experience of this type of labelling. The internal political debate in the European Union is still at an early stage and consensus has not been reached due to diverging views. The patterns that emerge from the analysis of the different point of views can facilitate the cooperation between stakeholders and policy-makers.

- Malnutrition points to the needs for a harmonized food labelling system in the EU
- Standardization in democratic institutions needs to include more stakeholders’ views
- Case study approach with focus on EU, Italian and Sweden perspectives
- A shared understanding of needs for a harmonized front package label
- Diverging views on the ideal labelling solution for the EU

## Introduction – needs for policy development

In Europe, the percentage of obese adults has been steadily increasing in the last decades and, under a business-as-usual scenario, around 37% of European adults will be obese in 2030 ([33]: 4). Krzysztoszek et al. [33] show that even if there are regional variation the incidence of overweight and obesity is high all over the continent. Furthermore, around 17% of European children are obese or overweight [28]. The malnutrition epidemic represents a social, environmental and economic problem for society [7, 34, 36, 41, 42].

Since the diet-related health outcomes are determined by several personal, social, economic, cultural and political factors, their precise influences are hard to measure [21]. In many cases, overweight and obesity can be prevented through a combination of individual and social measures that help people adopt a caloric intake that is adequate to their lifestyle. Even if the final decision of what to put on the plate is personal, it is influenced by a number of factors, such as price and access to nutritional information- for which businesses, public authorities, governments and NGOs are held accountable [45].

Over the years many EU countries have implemented voluntary front-of-package nutrition labels (**FOPNLs**) as part of national strategies to reduce diet related diseases [16].^1^ The FOPNLs used in the EU countries take into account different criteria and are thus not completely equivalent to each other, which may represent an obstacle to food-trade inside of the European single market and to Europeans’ understanding and use of these labels. Table 1 provides an overview of the FOPNLs that have been enforced across the EU.

**Table 1:** Examples of current front-of-pack nutrition labels on the EU markets

***Label name***	***Label illustration (example)***	***Country and year of adoption***
Keyhole		Sweden (1989); Denmark (2009); Lithuania (2013)
Nutri-Score		France (2017); Belgium (2019); Spain (2018); Germany (2020); Luxemburg (2020); the Netherlands (2019)
Heart Symbol – Better choice		Finland (2000)
NutrInform Battery		Italy (2020)
Healthy Living		Croatia (2015)
Protective Food -Little heart		Slovenia (1992)
Choices Logo		The Netherlands (2006- 2016); Poland (2008); Czech Republic (2011)

The European Commission aims to select and propose a single mandatory FOPNL to use in the entire EU by the last quarter of 2022. This is seen as part of an effort to restructure sustainably the whole EU agri-food landscape, as expressed in the Farm to Fork Strategy [15].

Different stakeholders have voiced contrasting opinions about the introduction of a new single FOPNL [13, 22–27]. The different positions of key stakeholders can challenge the development and implementation of a label if not addressed correctly. Even if governments should be the primary actors undertaking actions to promote public health, the discussion with industrial stakeholders can be beneficial if properly managed and aiming to sustain evidence-based approaches [35]. These public-private partnerships are based on the belief that association with the industry leads to better results than authorities acting autonomously does ([35]:6). Since possible conflicts of interest can arise there are contrasting views whether public private partnerships can be part of a strategy to tackle health issues through food policies [1, 31, 35, 38, 44]. If properly managed the criticisms of key stakeholders could lead to the development of better FOPNL that helps customers make healthier food choices. If poorly managed these partnership could significantly slow down or halt the development of a solution [31] despite the uptake of policies fostering public health should be done faster [8]. Phulkerd et al. [38] point to factors that can influence the development of a FOPNL positively or negatively such as type of monitoring system, clarity of the policy content, public knowledge, political priorities, and organizational aspects of the process. A dialogue-platform that brings together multiple stakeholders can be used as a way to tear down those barriers that might slow down policy implementation, while strengthening stakeholders’ relationships and finding better solution to the common issue (ibid.).

The aim of this paper is to identify the themes of the debate regarding the development of a single Front of Package Nutrition Label in the European Union. The following research questions (RQs) are in focus:

- *RQ 1. What are the opinions of different key stakeholders regarding a unified Front of Package Nutrition Label?*
- *RQ 2. What are the implications of the different views of a Unified Front of Package Nutrition Label?*
- RQ 3. *How do the European Institutions manage the process of developing a harmonized food label?*

## Theory

A theoretical framework has been developed to analyze the phenomenon, harmonization of a FOPNL. It starts with a description of standards and concludes with a conceptual framework created for the purpose of the research.

### Standards for harmonized communications

There are multiple definitions of the term standard, each of which highlight slightly different aspects of the concept and show that standards are used in regulating a variety of topics to enable coordination and cooperation [5, 9, 10, 37, 47]. Most of the definitions share common features about what standards represent in various contexts: standards are explicitly formulated, communicated and their use is voluntary. It also means that a standard must be perceived as valuable in order to be enforced by an individual or organization. However, a standard can also be implemented because of the pressure from third parties [10] or as a result of globalization [9]. Standards are also meant for the wide public, they are not developed by and for just a single user (ibid.).

**Fig. 1:**
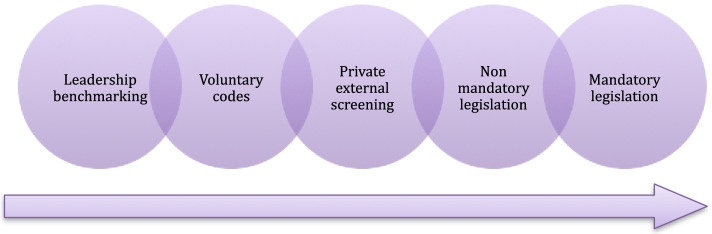
The Standard Spectrum shows how a good performance may lead the way for the standardization of a procedure (modified from Zadek [47]

Standard development may start as leadership benchmarking: an organization does something very well and that is picked up by others as efficient or as the best way to do things. The gradual change in procedures may find alternative roads. Some may lead to the development of legislation while others remain as voluntary codes. (Zadek [47], 3) offers a standard spectrum process that relates different expressions of standardized procedures to each other (Fig. 1). It is worth noticing that the standard formats to the right in the illustration influence more organizations and individuals.

There are different motives for standardizers to develop a standard, common denominators being the willingness to reach goals and solve problems [9]. Certain standards, such as the one set by the Forest Stewardship Council, are developed through a continuous stakeholder involvement. These standards are usually used for issues where the usual state-based legislation is lacking or not effective, and the actors involved are numerous and globally dispersed. Multi-stakeholder standards are often used to deal with social or environmental topics [2]. The involvement of stakeholders can lead to positive results, as it builds agreement between the parts and gives legitimacy to the standards (ibid.). In organizations governed democratically, the multi-stakeholder approach to standardization gains even more importance and therefore it becomes a fundamental part of the development [9].

Legitimacy is grounded in the credibility and acceptability of an accreditor. In polycentric regulatory regimes, such as the EU, there are additional issues to legitimacy building. These issues are connected to the coordination of the parts of the process, fragmentation of legislation, lacking clarity of who has authority, and different perception of what an “optimal” outcome looks like. All of these issues create a more complex setting for legitimacy building and regulation ([4]: 4-5).

The legitimacy to exert power over the standardized phenomenon is given to the standardizer by those adopting the standard and by those adapting their purchasing habits based to the standard, manifested in a visual symbol, an eco-label [2]. When an organization or person decides to use a standard, it gives the standardizer legitimacy to act and exert its power. Compared to mandatory rules, standards usually lead to less opposition, since those who are unsatisfied with a standard can simply stop using it. In practice, however, standards might not be as voluntary as they appear, due to institutional expectations of following certain standards. In addition, governance is also exerted through market power structures and financial incentives, not just through standards (ibid.).

### A context bound development of an EU food label

The conceptual framework for understanding conditions for the development of a harmonized FOPNL in the EU is based on Zadek’s spectrum [47]. It offers a contextual understanding of a process where key stakeholders take roles in different phases of the process. These stakeholder roles are divided into standardizers and other stakeholders. In this project, the standardizers are driving the standardization development process whilst other stakeholders may influence the development and be subject of its enforcement should it become legislation.

A standard with a third-party audit grants a label that can be used for communication [9]. The label is assumed to be the visible and instrumental part of a standard, since it is the part that all the stakeholders see. It is seen as grounds for potentially changing consumer behaviors and, consequently, the corporate conduct of the food industries and other stakeholders [3]. Standards are set through the process of standardization inside of boundaries defined by the laws. The process is driven by the standardizers, but the dialogue with the possible standards adopters and other stakeholders can also play a significant role, as they can give feedback that influence what each standard represents [2, 9]. Finally, the FOPNLs and the standards behind are moving towards the right of Zadek [47]‘s Standard Spectrum (Fig. 1). At the moment, they are set by non-mandatory national legislations but the EU goal is to make one mandated by common law.

**Fig. 2:**
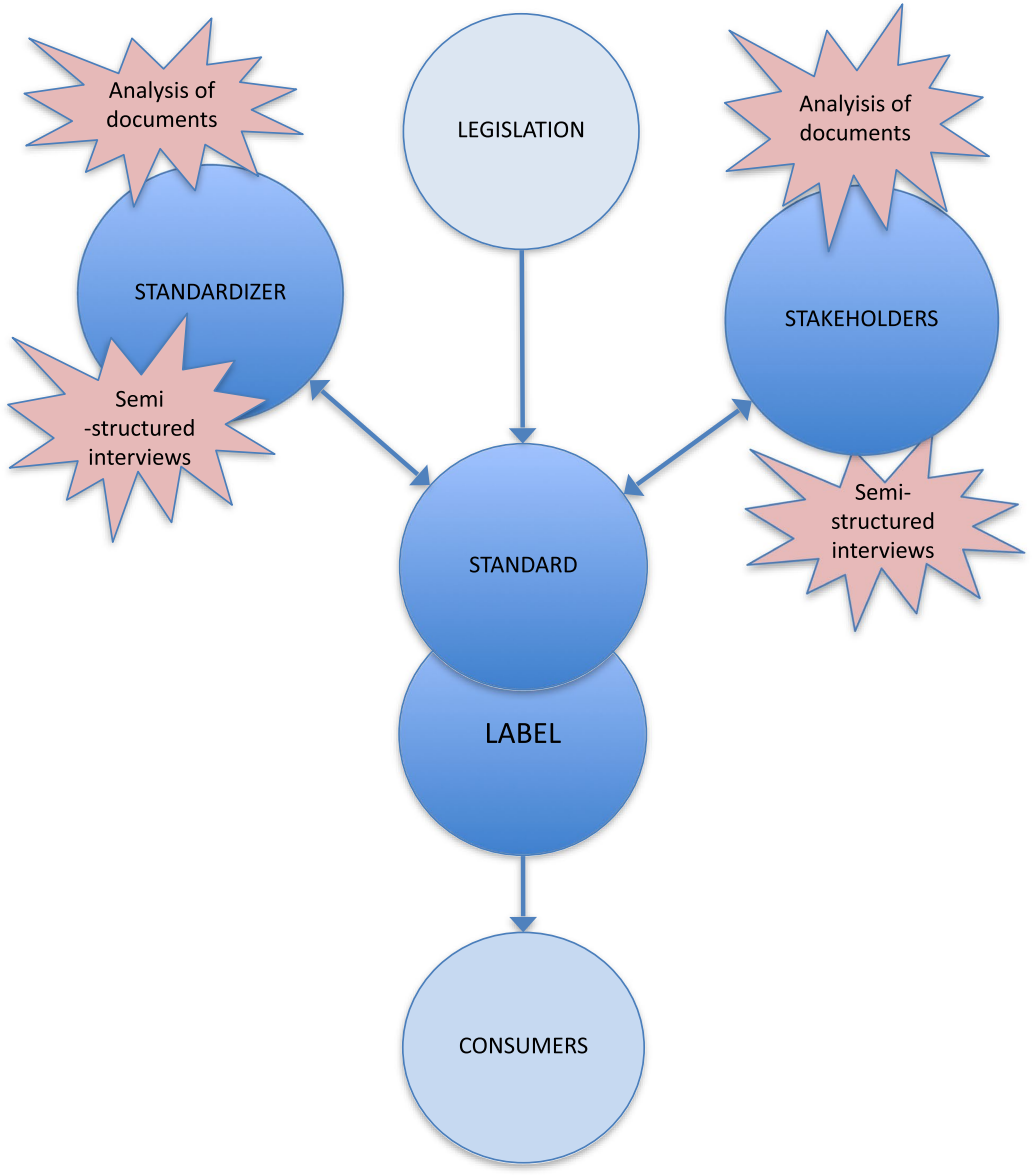
A conceptual framework for analyzing labels and standards. The label and the associated standard are created and managed by a standardizer, which is influenced by the stakeholders. Legislation and consumers, significant for the label and standard implementation, are not going to be considered for the scope of the project and are thus “cut-off” by red lines. The label and standard are seen as moving inside Zadek [47]‘s standard spectrum process

A visual representation of the context for the development of a European FOPNL is presented in Fig. 2.

The focus in this project is placed on themes of the debate regarding the development of a single FOPNL in the EU. This communication is an ongoing process where standardizers drive the dialogue with other stakeholders. In Fig. 2 the part between the red lines illustrate the focus of the study. Delimitations are made concerning the role of media and individual consumers, despite the importance of their role in communication. The European and National legislative frameworks that enable and control the existence of FOPNLs are not included in this project.

## Methods

This project has a unit of analysis that is represented in the dialogue of the development of a food policy standard in focus. It is what (Yin [46]: 40) refers to as a revelatory case. The theme for the revealed case is the development of a united European FOPNL. It is presented as a case study, allowing for context bound understandings from two member states’ perspectives, Italy and Sweden. These two member states were selected as they have very different experiences with FOPNLs. Sweden was the first European country to implement a FOPNL in 1989, known as the The Keyhole. The label is widely used and familiar to population and industry. Italy, on the other hand, had no official FOPNL until the late months of 2020 and the new NutrInform Battery is still largely unused an unknown. Public and private actors are engaged in the development of FOPNLs in both countries. The cultural settings of Sweden and Italy differ in terms of dietary traditions and legal governing frameworks. Despite these differences, participation in the European Union requires willingness to find common ground in a variety of sectors in order to achieve joint policy goals. Because the Swedish and Italian experiences with FOPNLs are so dissimilar, it is reasonable to assume that the other Member States’ debate will mirror the one that Italy and Sweden reflect.

### Data collection

Data were collected using secondary data (policy documents) and primary data in semi-structured interviews. Ethical guidelines were followed concerning the use of work in progress documents for policy development, informant GDPR and research conduct in general [40]. This is particularly important given the political nature of the project.

The selection of policy documents of interest for the FOPNL development was made in the web pages of central organizations. The text, in English, Italian and Swedish had the form of web pages, letters and documentation of dialogues. Semi-structured interviews with representatives of key organizations of importance for food policy development (Table 2) served as a second step in the collection of empirical material. These interviews also gave rise to insights to make a continued policy document analysis.

**Table 2:** Outlook of key stakeholders

Stakeholder group	Role	Sweden	Italy
Association of consumers	Non standardizing	Swedish Consumer Association	Italian Consumer Association
Association of Food Industries	Non standardizing	Swedish Food Federation	Italian Federation of Food and Drink Industry
Association of retailers	Non standardizing	Swedish Food Retailer Federation	Italian Trade Business Federation
State Agency	Hybrid	Swedish Food Agency	Health Ministry
EU Commission	Standardizer	Member State	Member State
EU Council	Standardizer	Member State	Member State

Table 2 visualizes the identified key stakeholder for each member country and their function, stakeholders with a similar function are on the same row. Different stakeholders have different roles in the standardization process. State Agencies have a hybrid role, as they are the standardizer on a national level, but might not be on a European one. Thematic interviews were carried out with representatives of the identified key organizations. It means that the researcher prepared a set of themes for questions that should be utilized during the interview, but the interviews were not bound to these *ex ante* identified questions, as suggested by (Robson and McCartan [39], 290-291). Because of the limitations imposed by an on-going COVID-19 pandemic, the interviews were conducted via the digital platforms such as Zoom and Teams. All the interviews were built starting from the same “question themes”: past and present experiences with the national label, the views on the need of a common European label, characteristics of the ideal label and type of involvement with the label development process. Despite the themes being the same, the exact wording differed depending on the stakeholder’s background. The representatives from each key stakeholder are presented in Table 3.

**Table 3:** List of the interviewees representing key stakeholders and the dates for the interview procedures

Key stakeholder	Type	Date	Summary sent	Validated
Swedish Food Federation	Interview on Teams	15/03/21	17/03/21	22/03/21
Swedish Consumer Association	Interview on Zoom	23/03/21	25/03/21	25/03/21
Swedish Food Agency	Interview on Zoom	23/03/21	25/03/21	29/03/21
Italian Consumer Association	Interview on Zoom	22/03/21	25/03/21	20/04/21
Italian Federation of Food and Drink Industry	Written interview	17/04/21	X	X
Italian Trade Business Federation	Phone call interview	31/03/21	1/04/21	1/04/21
European Commission / Health and Food Safety / Office E1: Food Information and composition	Interview on Teams	19/04/21	20/04/21	X

Table 3 shows the list of interviewees for each key stakeholder, how and when the interview was conducted and validated. Validation was done in two steps in the research procedures, during the interview in confirmatory techniques and in sending the respondent a summary of the dialogue, which they were able to modify so to better represent their position.

### Data analysis

The empirical material, policy documentation and interview transcripts were used in a thematic framework analysis approach that had been established as useful [39]. The iterative process is reflected in comparisons and contrasts of documents and interview transcripts from the two countries. The perspective of the EU Health and Food Safety represents shared EU perspectives. Emerging themes in the analytical process had theoretical grounds, from the literature review that gave rise to the themes for the interview, as well as purely empirical grounds from the documents and interviews.

It is worth keeping in mind that the policy development process is a longitudinal process. Interviews and policy documents from a particular time may not justify the process as a whole, even if efforts have been made to ask about the development process as a whole. Respondents are expected to account mostly for recent and current developments.

## Results

This section focuses of the results of the analysis of documents and interviews. The results coming from the non-standardizing stakeholders and the ones from the standardizer are presented in subsections. It is the standardizer, the EU, which owns and runs the development process.

### Non standardizing-stakeholders

The results in this subsection are further divided in two parts. The first section deals with the debate around the label itself, while the second deals with the opinions about the standards behind the label.

#### Desired features of an harmonized label

**Fig. 3:**
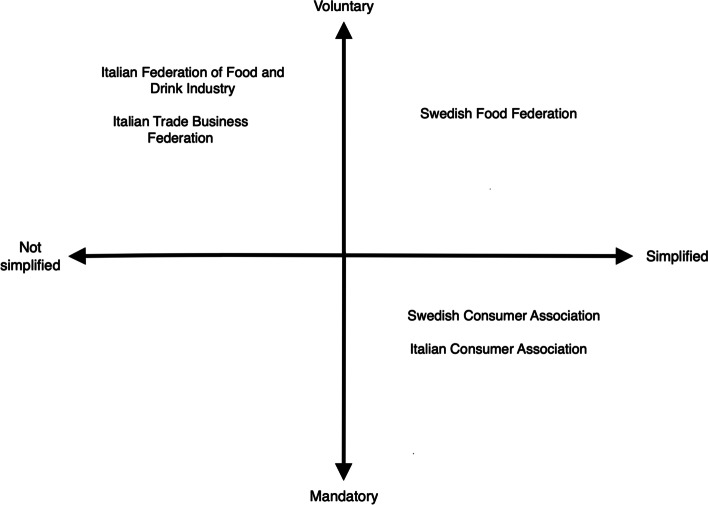
A map of the stakeholders positions in relation to some desired features

The interviews show that different stakeholders have different expectations of how the common FOPNL should look like to consumers. The positions for key stakeholders with regards to voluntariness and simplified format are illustrated in Fig. 3 and the desired features from interviewed stakeholders are presented in Table 4.

**Table 4:** Desired features of the “ideal” food label from different stakeholders’ views

Some of the desired feature in the label	Stakeholder(s)
Voluntary	Swedish Food Federation; Italian Trade Business Federation; Italian Food and Drink Industry Federation
Mandatory	Italian Consumer Association; Swedish Consumer Association
Simplified information	Italian Consumer Association; Swedish Consumer Association; Swedish Food Federation
Non-simplified information	Italian Trade Business Federation; Italian Food and Drink Industry Federation
Based on portion size	Italian Food and Drink Industry Federation
Non discriminating	Italian Food and Drink Industry Federation; Swedish Food Federation; Italian Trade Business Federation

These illustrations (Fig. 3 and Table 4) show that a number of the desired features are contradictory and hard to find middle grounds for in the development of a shared FOPNL (RQ1). The representatives of the two Associations of Consumers agree that such a label should be simplified, based on colors that show the full spectrum of grades (from positive to negative) and mandatory. The representative of the Italian Association of Consumers thinks that consumers would more easily use a label with those characteristics, as it would enable them to compare products without too much effort; they also think that the label’s efficiency to change patterns of consumption would depend on the label’s diffusion on the market.

Representatives of the food industries, however, have different opinions on the features of the “ideal” common label. The Italian Business representatives question the attributes wished by the Consumers Associations, while being in favor of a label that is not simplified, or based on colors, arguing for a label that presents more information about the single nutrients. The label should also be voluntary and non-stigmatizing. The Swedish Food Federation agrees and stresses the importance of a voluntary label, stating that if the label works properly and is appreciated by the industries, then it is going to be adopted anyway. Similarly, the Swedish Food Agency brings up the different capacities among small and medium enterprises of the food system to find the resources to adapt to a mandatory FOPNL, which leads to a preference of the voluntary scheme. The Swedish Food Federation is in favor of an interpretative scheme. They support positive and non-stigmatizing solutions, but some members now support interpretative schemes with a range of grades.

With these very contrary views on the ideal FOPNL in mind, the next step is to consider the rules and criteria behind the label.

#### Desired features of the harmonized standard

The principles of desired features of a shared FOPNL are expressed in this section with connection to the empirical context and the role that the stakeholder representatives have. The label features are presented in Table 5 (RQ2).

**Table 5:** Desired features of the “ideal” standard from different stakeholders’ views

Some of the desired feature in the standard	Stakeholder(s)
Clear link with national dietary guidelines	Swedish Food Agency; Swedish Food Federation
Account dietary tradition	Italian Trade Business Federation; Italian Food and Drink Industry Federation
All food categories covered	Italian Trade Business Federation; Italian Food and Drink Industry Federation; Italian Consumer Association
Nutritional profile	Italian Consumer Association; Swedish Consumer Association; Swedish Food Agency; Swedish Food Federation
On 100 g or milliliters	Italian Federation of Food and Drink Industry

The representatives of Swedish Food Federation and the Food Agency argue that the nutritional criteria behind the label should always have a clear link with the national dietary advice, which also include food traditions. The Italian Association of Consumers, however, suggests that the differences between national dietary guidelines are not so significant and the nutritional issues are mostly the same all across the EU. The representative of the Italian Businesses did not mention the national nutritional guidelines, but argue that any scheme should take into account dietary tradition.

The issue of which food categories should be covered by criteria, and thus possibly granted a label, is also a source of disagreements. Although they strongly disagree on how the ideal label should be visualized, the Italian stakeholders agree that the label should be on all product categories. The Italian Representatives of Businesses state that all products should be given the possibility to bear the label, as all foods can form part of a balanced diet formed by appropriate quantities and frequencies and combined with physical activity. The representative of the Italian Consumer Association is also in favor of applying the label on all food categories. In the case of a label with a graded indicator, they specify that products granted a negative rating should not be completely avoided but instead consumed with moderation.

On the Swedish side, however, some voices favor the exclusion of certain food categories. The representative of the Swedish Food Agency argues that the label should only be applied to the core-foods, those that the population consumes the most, and should avoid giving a positive image of foods that are not in line with the dietary recommendations. The representative of the Swedish Association of Consumers has a less clear position, they think that most categories should be covered by criteria, but could accept that some categories are not covered. They are skeptical about expanding criteria to all foods as some consumers with totally wrong diets could think they are following healthy diets just because they are eating the “labeled-segment”.

The parties that oppose an interpretative label, such as the representatives of the Italian Business Associations, say that the label should provide “un-filtered” information about the nutritional content of the food the label is attached to. As a consequence, there are no nutritional profiles behind the display of the label. However, the actors that favor an interpretative label also raise the question of which criteria should be satisfied in order to reach a certain “grade”. All the stakeholders agree that this negotiation is going to generate heated discussion. The Swedish actors highlight that the criteria are also strongly influenced by the national food habits and that establishing common criteria for the Keyhole had been challenging even in the Nordics, where the food habits are more similar than across the other European countries. The representative of the Swedish Food Agency also specifies that the criteria should take into account the main nutritional issues in the target areas.

Two Italian stakeholders also raised the issue of whether the label should be based on 100 g/millilitres or on portion size. The representative of the Italian Federation of Food and Drink Industry stresses that the label should present information based on the content of nutrient based on a portion of that food, as that better represents the nutrient intake actually associated with that food. The representative of the Italian Association of Consumers, however, argues that there are still no existing standardized-portions, and thus in the present conditions referring the label to a portion could mislead consumers.

### The standardizer

The standardizer, in addition to the non-standardizing stakeholders, is analyzed to better understand the process (RQ3). This sub-chapter focuses on the standardizer, represented by the EU. In this phase of the legislative process, the Institutions playing a bigger role are the European Commission and the Council of Ministries. The following paragraphs present their current stance regarding the harmonization of FOPNLs.

#### European Commission

At the moment, the Commission is gathering the existing scientific evidence about how FOPNLs work, so to support the decision making process that will lead to a proposal for a harmonized food labeling plan. An initial consultation round was already done as preparation for the Inception Impact Assessment^2^ (**IIA**) [14]. A number of Swedish and Italian stakeholders have taken part to the open consultations [17–19]. The feedbacks on the document represent position in line with what presented in the interviews of this project. In the proposal process, further evidence, from a more systematic collection, will be contained in a specific Impact Assessment (**IA**), which is also going to be associated with an extensive round of consultations with stakeholders, panels of experts, citizens, etc.

In 2020, the Commission’s Centre for Policies Report published a work presenting state-of-the-art knowledge about FOPNLs, but as new evidence emerge a new updated report will be produced. Furthermore, the European Food Security Authority (**EFSA**) is going to help identify which are the nutrients of public health relevance in the EU, which food groups play important roles in the different cultures of the Union and the criteria to be covered or not covered by the future labeling scheme [12].

These scientific reports are going to help expand on the issues connected with FOPNL schemes, which were also mentioned in the interviews with the other stakeholders, in the IIA and in the feedbacks. However, even if these reports will be included in the Impact Assessment, they will not determine what will be written in the proposal, as they only provide reliable information to the Commissioners, which are the ones responsible for making the decisions. Still, the aforementioned scientific evidences will probably help to bridge between the different parts in the political debate and find common ground.

The stakeholders in Italy and Sweden have presented contrasting views whether the label should be voluntary or mandatory, as stated in the Farm to Fork Strategy. The interviewees from the Commission stated that the Commissioners have put forward a mandatory label, as it seems the best response to citizens’ request for such a label. If a mandatory harmonized label is to be established, the current rules will also have to be changed. The Initiative is, in fact, called “Proposal for a revision of Regulation (EU) No 1169/2011 on the provision of food information to consumers” [14]. However, even this label’s feature is still up to discussion.

#### Council of the European Union

The Agriculture and Fisheries Council has been discussing a draft of the Council Conclusions on FOPNLs during some of its meetings. Even if the meeting occurs on a monthly basis, discussion about the harmonized FOPNL in mid-December 2020 [20] did not lead to consensus (ibid.). The Council’s Conclusions were not adopted because of the sole opposition of the Italian, Greek and Czech National Delegations.^3^ It was not possible to attain official documents to resolve the disagreement, but some of the possible issues can be supposed by comparing the content of the proposed Conclusions with the one of a non-paper document^4^ sent in September 2020 by some of Delegations to the Council.

Noticeably, some of the issues raised by the Delegations in September were not addressed in the proposed Conclusions text and thus might have created the political misalignments that blocked the Conclusions from passing. However, some of the points were addressed and might have led Cyprus, Hungary, Latvia and Romania to become supporter of the Conclusions, while still not convincing the Italian, Czech and Greek Delegations. Table 6 provides the reader with a brief comparison between the issues addressed by the two documents. It highlights common grounds and differences.

**Table 6:** Political convergences & divergences inside of the Agriculture and Fisheries Council

Issues	Signatories of the Presidency Conclusions on FOPNLs – December 2020	Signatories of the non-paper on FOPNLs - September 2020
Harmonization	Yes	Yes
Complementary to national nutrition guidelines and respectful of national cultures	Yes	Yes
Easy to understand without in-depth nutrition knowledge, visible and unambiguous	Yes	No
Transparent for the consumers and easy to monitor	Yes	No
Exclusion of certain food categories (such as PDO, PGI, TGI and single-ingredient products)	Yes	Yes
Non evaluative label, no use of colors but provision of factual information	No	Yes
Reference to actual intake instead of 100 g or milliliters	No	Yes

As Table 6 shows, while all Member Countries unite on certain characteristics the labelling scheme should have, there are still huge differences of views over other issues, such as evaluative or non-evaluative labels and reference on a portion instead than on 100 g or millilitres. Some of the fractures inside of the Council reflect the differences in opinion regarding the harmonized label that arose during the interviews with the stakeholders.

## Discussion

The present study addresses the issue by investigating what the current debate on a harmonized FOPNL in the EU is about, so that a way forward can be identified. In fact, studies like Breda et al. [8] show the necessity of accelerating the uptake of policies sustaining the transition towards a healthier society. In particular, there is the need for a fast and wide uptake of policies establishing a FOPNL that can be easily used by consumers (ibid.). Phulkerd et al. [38] pointed out that the establishment of multi-sectorial platforms provides meeting ground for stakeholders and policy-makers that could contribute to the prevention of barriers further down in the label development process. The lack of such platforms, together with the opposition of the food industry to new labeling, represent two of the main obstacles to the development of a new FOPNL (ibid.).

The next subchapters discuss the results in relation to all the stakeholders, followed by the political and research implications of this work.

### The non-standardizer stakeholders’ debate

As expected, the points of view of different stakeholders on a single European FOPNL strongly differ from one another, creating a debate dealing with virtually all the features of a common labeling scheme. The positions reflect the difference in interests represented as well as experience with the national FOPNL schemes. Creating common criteria will be about balancing the needs and expectations of consumers with the needs and expectations of producers. Different stakeholders expect the new FOPNL to guide consumers towards healthier choices while incentivizing the industry to reformulate. We can expect that the more stakeholders at the negotiation table, the harder it gets to find common grounds for decisions. As new scientific evidences are available, new positions need to be negotiated in the process.

Some interviewees use arguments or actions against an evaluative FOPNL that are similar to those used by certain stakeholders during the development of the French FOPNL and described by Julia and Hercberg [31]. The actions of the French food industries reflect reaction on a proposed FOPNL developed by the French Minister of Health (ibid.). The French representative of food businesses argued that such a label was potentially discriminatory and based only on a simplistic and functional approach to food, while arguing that the label should take into account the whole setting in which food consumption actually takes place. As the Italian Representatives of businesses, the French also worked together to develop their own label, which was not supported by science in all its features and was criticized for being complicated for consumers to understand (ibid.). Eventually, these actions slowed down but did not stop the development of a FOPNL in France. The actions undertaken by the French food businesses were eventually uncovered by the press and have led to a negative public opinion towards them (ibid.). Temple [44] also reports the lobbying actions that the agri-food industries have carried out in the United States and that have led to set-backs in the creation of policies aiming to improve public health through policies impacting diets. These experiences highlight the importance of managing the dialogue properly to support the implementation of the harmonized FOPNL.

The resistance of certain industrial stakeholders to public-private solutions addressing public health has led many authors such as Moodie et al. [35] to conclude that those industries producing unhealthy food should not play a role in the development of related legislation. Moodie et al. [35] conclude that government, while discussing with stakeholders, should always be grounded in scientific evidence. However, the experience of the Keyhole proves that stakeholders with very different agendas can collaborate on initiatives promoting public health. The Keyhole collaborative model could be used as a template for managing the debate between the standardizer and the non-standardizing stakeholders. Still, the different cultural background of the stakeholders, of the consumers and other regional differences might undermine the capacity of the model to be transferred. These cultural differences are still significant in the EU [30]. In any case, with proper management, it may be possible to obtain a quicker and smoother development of a harmonized European FOPNL, which ideally would create a new equilibrium for all the parts of the food system and a healthier society.

The analysis of the case shows that all the represented stakeholders advocate their views both on a National and International level, in an ongoing dialogue between them through national tables or European Associations of Categories. This is in line with what is reported by Balzarova and Castka [2]. They noticed that the stakeholders that are more active, for example producing a bigger amount of comment to the standard’s draft, are also going to be more influential. The research showed also that the comments were accepted more often when falling in their areas of expertise (ibid). At the current phase of development of the harmonized labeling scheme it still unknown which points of views will be integrated in the Proposal to be discussed. However, the current activity of the stakeholders reveals their willingness to be heard and influence all aspect of the future labeling scheme.

Balzarova and Castka [2] identified steps that stakeholders follow in order to influence and contribute to the standard debate. At the moment (summer 2021), the contributions from the stakeholders to European FOPNL development is still in at the elimination and linking phases of the process, even if comments about themes that will be further discussed are already being made.

This project confirms the understanding that voluntary solutions could raise less resistance during their establishment, as argued by Temple [44]. Voluntary schemes are more likely to be adopted and, even if their effects would be less pervasive than those of a mandatory solution, they could still bring to positive results by highlighting the healthiest products to consumers and incentivizing reformulation efforts [44]. Still, it unclear how these communication efforts could finally impact actual consumer purchasing and dietary choices [43].

### Standardizer and standard development

The European Council is still at an early stage of the Proposal’s development, but the competent offices are developing the scientific materials that the Commissioners will use to the make their decisions. In addition, the opinions of different stakeholders have been collected as feedbacks to the IIA and will be further investigated into the IA. This is in line with Balzarova and Castka [2]‘s finding that the standardizers tend to reach a consensus between the stakeholders by including their opinions in the process. The views of the stakeholders are also used as a way to keep the standard’s focus on real issues and control its effects [5, 9]. At the same time, the efforts being done by the European Union to collect evidence and opinions from the stakeholders resonate findings by Black [4]. It is worth noting that, actions that might make an organization more legitimate for an actor might make it less legitimate for another (ibid.), in particular when the stakeholders have contrasting interests, as in this case study.

In line with the literature [9], the stakeholders pointed to globalization as the main reason for a harmonized FOPNL; goods are sold to other countries in the single market and consumers travel. A less fragmented solution which still preserves the national differences would be appreciated by many. Since there is not a global formal organization and legislative body, standards can represent a way to coordinate these actors. For example, the European Union has little authority since its members are nation-states that want to preserve, with different degrees, their independence. Standards are thus used as an alternative mean of governance, since the member countries and the single organizations perceive them as voluntary. Supposedly, a mandatory labeling scheme would create more divergences than a voluntary one would do.

Nonetheless, there are also obstacles to the homogenization of standards across international borders, such as the high costs of changing regulatory systems that are already in place and the fact that international standards might be adaptable to the specific needs of certain countries, etc. [6, 29]. These obstacles were also reflected in the words of the stakeholders that, for example, are worried about losing a well-established local label or argue that it is hard to get over national dietary differences.

Finally, when it comes to a transition point of view, the European FOPNLs are moving inside of Zadek [47]‘s Standard spectrum. At the moment they are set by non-mandatory legislation but, should the development process lead to a mandatory solution, the new label would be set through mandatory (international) legislation. Should a new harmonized FOPNL be made non-compulsory, it would still be an example of voluntary (international) legislation impacting a bigger number of industries, businesses and consumers.

Given the complexity of research, there is limited evidence of the precise effects of FOPNLs on purchasing behaviors, diet shifting and overall health [43]. However, the research available shows that FOPNLs can be a tool that help people adhere to the dietary recommendations while pushing the industry to reformulate towards nutritionally better foods (ibid.) The effects of eating patterns on collective health are only visible overtime and the precise relationships are hard to single out, showing the need of synergic approaches in which the different actors in the food system work in the same direction. The synergy begins with an effective dialogue between the stakeholders, supported by scientific evidence. As this paper shows, the dialogue regarding FOPNLs is ongoing but, given the variety of opinions presented, it is far from reaching a solution. A failed agreement can lead to a FOPNL that is not aligned with the broader dietary recommendations, that is not picked up by consumers or industry, that is not effective or understood and that finally impedes healthier food habits.

### Policy implications

As a consequence of differences between the stakeholders a harmonized FOPNL has been politicized from the very beginning of the law-making process, when all the features are still to be defined, possibly slowing down the implementation process. Although the proposal is not written yet, both on a European and National level statements are being made about what it should or should not contain. However, even in this early phase of the process the Commission has to think about the political views, as the politicians in the European Parliament and Council will eventually discuss any proposal.

As the present work shows, finding mutual ground in the legislative process represents a set of challenges, especially when the expected stakeholders have different agendas and experience. Still, doing so is fundamental in democracies. A systematic collection and comparison of the different point of views is expected to benefit both the stakeholders and the politicians and contribute to the development of an appropriate governance structure, as expressed by Phulkerd et al. [38]. Politicians would get a clearer vision of the consequences of any decision they may take while, by satisfying as many stakeholders as possible, they would also gain legitimacy and support. By systematically analyzing the debate, the stakeholders can compare their views to those of others, find allies and eventually produce comments that are more likely to be accepted and influence legislation making. The systematic management of the debate could make faster the legislative process, as called for by Breda et al. [8], while limiting the risks connected to stakeholder involvement in public health policies [35].

While the details of the different points of views are entrenched in their national contexts, the general elements of the debate emerged from this study could be of relevance for stakeholders in other Member States or non EU-countries that want to implement this type of labeling scheme or other public health policies. Still, the cultural dimension of this political process should not be underestimated. It points to the need of multi-cultural ability to comprehend the different perspectives and find shared grounds and meanings. The knowledge gained with this study also has application in the fields of standard or policy development and stakeholder management, for a set of shared resource management tools in the member states.

### Research implications

The present research illustrates the debate around the development of a European FOPNL in a short period of time and inside of a geographically limited area. This limits the possibility of generalizing the results, but also does not enable the writers to follow the debates overtime as decisions, are taken. At the same time, is yet not possible to know which stakeholders views will be incorporated into the Commission’s proposal and final legislative text. Future research could investigate the FOPNLs debate in other countries and over a much longer timeframe, so to see its evolutions as agreements emerge and decision are taken. Studying the development of a FOPNL over a long period of time would also shows which stakeholder’s feedbacks are included in the final standard and which are not. Research could focus on the development of other types of labels, such as environmental ones, to see if the type of information changes the debate or if the themes are the same. Finally, future research could follow up on other type of policies, especially those involving health and environment, to see how stakeholders’ opinions are framed to managed and to which the final policy outcomes they lead. Finally, future research could expand the understanding of how standards in international regulation.

## Conclusions

This project contributes to the understanding of themes in the debate regarding the development of a single FOPNL in the EU. While the stakeholders agree on the benefit of label harmonization on a European level, they have very different opinion with regards to the features the the new labelling scheme should have. The disagreements emerge on the lines of the role of the stakeholder and of its country of origin and reflect the different national experiences when it comes to FOPNLs.

The stakeholders are participating in various ways to the development of their national labels, while trying to influence the European process. Their point of views and experiences are reported to the policy-makers directly or through the mediation of European associations of category.

This project aimed to identify the themes in the ongoing dialogues concerning FOPNL. The themes currently debated among the stakeholders or inside of the European Institutions are:

- Goal of the label: to inform consumers *or* to guide them
- Type of enforcement: voluntary *or* mandatory
- Type of design: evaluative label or not, scale of grades *or* positive character
- Food categories: type of divisions, exclusions, nutritional profiles and criteria
- Degree of regionalization: link with dietary guidelines, food traditions, and regional health issues
- Quantity the label refers to: on 100 g or millilitres *or* portion-based

Currently, the process developing a harmonized labelling scheme is at an initial state inside of the EU Institutions. The European Commission has stated, in the Farm to Fork Strategy, with the intention of proposing such a label in the last quarter of 2022. The Commission has published an IIA, which citizens and stakeholders in the EU weere invited to comment, and is currently working to collect the relevant scientific evidence that the Commissioners will eventually use in the Proposal. The European Council of Ministries has also already been discussing the topic, but the Ministries have not yet been able to reach a consensus on which features the labelling scheme should have. For the most part, the political debate in the European Council reflects the one presented by the stakeholder interviewed and is expected to continue to evolve until a final agreement if reached.

Suggestion for future research are based on the understanding of needs for a systematic management of democratic dialogue in order to develop shared policies for public health. Continued research needs to identify enabling factors for democratic policy development, for the process of policy development as well as for its implementation. Sustainability challenges of all sorts point to needs to coordinate policies globally in various incentive structures where standards may serve as an important step towards mandatory legislation. The present paper also points to the need for further research at the debate in other member states and over time, so to understand how the different opinions will be integrated in the final policies.

## Data Availability

The data used are the authors own and were collected in the first semester of 2021.

## Footnotes

1 FOPNLs are present information on the front-side of a package and are more efficient than back-side ones in shifting people’s buying intentions, as they are more easily spotted by the potential consumers (Kelly & Jewell 2018 [32]; Campos et al. 2011 [11]). Still, there are knowledge gaps regarding their effects on people behaviors and health [43].

2 An Inception Impact Assessment is a document produced at the very beginning of the legislative process, which aims to inform citizens and stakeholder on the Commission’s legislative plans, so to allow them to provide feedbacks that can help that deepen the Commission’s understanding of the problem and the possible solutions and impacts. The document provides a context to the proposed policy; briefly illustrating the problems it aims to solve, the policies objectives and options, and an assessment of the impact the policy would have on economy, society, environment, fundamental right and on administrative burden. The document also presents some of the scientific evidence behind the policy. Once the IIA is published, open consultation are held and, afterwards, an Impact Assessment is published (European Commission, 2021)

3 Presidency Conclusions on Front-of-pack nutrition labeling, nutrient profiles and origin labeling. Sent from the Presidency to the Delegations on the 15 December 2020

4 Front of Pack Nutrition Labeling – *Information from the Italian and Czech Delegations, on behalf of the Cyprus, Czech, Greek, Hungarian, Italian, Latvian and Romanian Delegation.* Sent from General Secretariat of the Council to the Delegations on the 17 September 2020.
